# 8-Year Long-Term Outcome Comparison: Two Ways to Exclude the Internal Iliac Artery during Endovascular Aorta Repair (EVAR) Surgery

**DOI:** 10.1371/journal.pone.0130586

**Published:** 2015-07-20

**Authors:** Han Luo, Bin Huang, Ding Yuan, Yi Yang, Fei Xiong, Guojun Zeng, Zhoupeng Wu, Xiyang Chen, Xiaojiong Du, Xiaorong Wen, Chuncheng Liu, Hongliu Yang, Jichun Zhao

**Affiliations:** 1 West China Medical School of Sichuan University, 37 Guo Xue Alley, Chengdu 610041, Sichuan Province, China; 2 Department of Vascular Surgery, West China Hospital, 37 Guo Xue Alley, Chengdu 610041, Sichuan Province, China; 3 Department of Ultrasound, West China Hospital, 37 Guo Xue Alley, Chengdu 610041, Sichuan Province, China; University of Washington, UNITED STATES

## Abstract

**Purpose:**

To evaluate the 8-year long-term outcome after internal iliac artery (IIA) coverage with or without embolization in EVAR.

**Patients and Methods:**

From January 2006 to December 2013, abdominal aortic aneurysm (AAA) subjects that underwent EVAR and IIA exclusion were recruited and analyzed retrospectively. All the subjects were divided into group A or B based on the presence or absence of intraoperative IIA embolization before coverage (group A: without embolization; group B: with embolization). The 30-day mortality, stent patency, and the incidences of endoleaks and ischemia of the buttocks and lower limbs were compared. The follow-up period was 96 months.

**Result:**

There were 137 subjects (A: 74 vs. B: 63), 124 male (91.1%) and 13 female (9.5%), with a mean age of 71.6 years. There were no significant differences in the early outcomes of intraoperative blood loss (87.23±14.07 ml; A: 86.53±9.57 ml vs. B: 88.06±18.04 ml, p = .545) and surgery time (87.13±9.25 min; A: 85.99±7.07 min vs. B: 88.48±11.19 min, p = .130). However, there were significant differences in contrast consumption (65.18±9.85 ml; A: 61.89±7.95 ml vs. B: 69.05±10.50 ml, p<.001) and intraoperative X-ray time (5.9±0.86 min; A: 5.63±0.49 min vs. B: 6.22±1.07 min, P<.001). The 30-day mortality was approximately 0.73%. In the follow-up analysis, no significant differences were identified in the incidence of endoleak (22 subjects; type I: A: 2 vs. B: 2, p = 1.000; type II: A: 8 vs. B: 4, p = .666; type III: A: 4 vs. B: 3, p = 1.000), occlusion (5 subjects; 4.35%; A: 1 vs. B: 4, p = .180), or ischemia (9 subjects; 7.83%; A: 3 vs. B: 6, p = .301). In the analysis of group B, although there were no significant differences between subjects with unilateral and bilateral IIA embolization, but longer hospital stays were required (P<.001), and a more severe complication (skin and gluteus necrosis) occurred in 1 subject with bilateral IIA embolization.

**Conclusion:**

IIA could be excluded during EVAR. IIA coverage without embolization had a good surgical and prognostic outcome, and this procedure was not different significantly from coverage with embolization in terms of endoleaks, patency and ischemia.

## Introduction

Since Dr. Parodi first developed minimally invasive endovascular technology in 1991[1, 2] to treat abdominal aortic aneurysms (AAAs), endovascular aortic repair (EVAR) has been the primary choice for AAA. Currently, nearly half of AAA patients undergo EVAR after IIA exclusion[3]. When the distant landing zone in the common iliac artery (CIA) is too short to anchor, the endograft limb should be extended into the external iliac artery (EIA), thus covering the original internal iliac artery (IIA). Although some researchers and doctors advocate preserving the IIA because it is a crucial collateral artery of the lower extremities and pelvis[4–7], excluding the IIA during EVAR has been proven safe, and it is an effective way to avoid endoleaks[3].

However, the methods for excluding the IIA have recently become controversial. Some studies suggest that IIA embolization with coils is much more effective than direct coverage with a stent in avoiding type II endoleaks[3, 8, 9]. However, other results show that excluding the IIA without embolization has a similar long-term outcome to the procedure with embolization[10–12].

Therefore, a retrospective cohort study was designed at our institution to compare the long-term outcome of two exclusion methods (absence or presence of a coil) and to evaluate the effectiveness of IIA exclusion. Here, we would like to report the results of our study.

### Patients and methods

The patients that were included in this study were recruited from the Vascular Department of West China Hospital, Sichuan University, between January 2006 and December 2013.

#### Inclusion criteria

In total, 702 patients with a diagnosis of AAA were included in this study. Cases of AAA were diagnosed via ultrasound or computed tomography (CT), and isolated cases were diagnosed by magnetic resonance angiography (MRA). All the repaired AAAs were more than 5.5 cm in diameter or showed rapid growth (>0.5 cm over 6 months).

#### Exclusion criteria

The exclusion criteria consisted of four parts: A, open surgery; B, unilateral or bilateral IIA(s) was (were) not excluded; C, instructions for use (IFU) were not followed. Three factors regarding the neck of the AAA were selected among the IFU as exclusion criteria, including the 1) length of aneurysm neck ≤15 mm; 2) angulation of infrarenal neck >60°; and 3) neck atheroma with a thickness and length >5 mm (measured via preoperative CT)[13, 14]. Patients who had one of the characteristics above were excluded from this study. D, Surgery time: emergency EVAR, such as EVAR for ruptured AAA (rAAA). Here, rAAA indicates an emergent unstable hemodynamic condition during surgery, which would negatively influence a surgeon’s decision whether to preserve the IIA.[15] Moreover, emergent EVAR is a life-saving surgery; therefore, surgeons would try to shorten the operation time to cover the aneurysm leak as soon as possible.

Based on the inclusion and exclusion criteria, 137 subjects were enrolled into the final comparison analysis. All the enrolled subjects were divided into two groups according to the presence or absence of intraoperative embolization IIA before coverage. Subjects without embolization were included in group A, and those with embolization were placed in group B.

### Internal iliac artery (IIA) management

**Fig 1:**
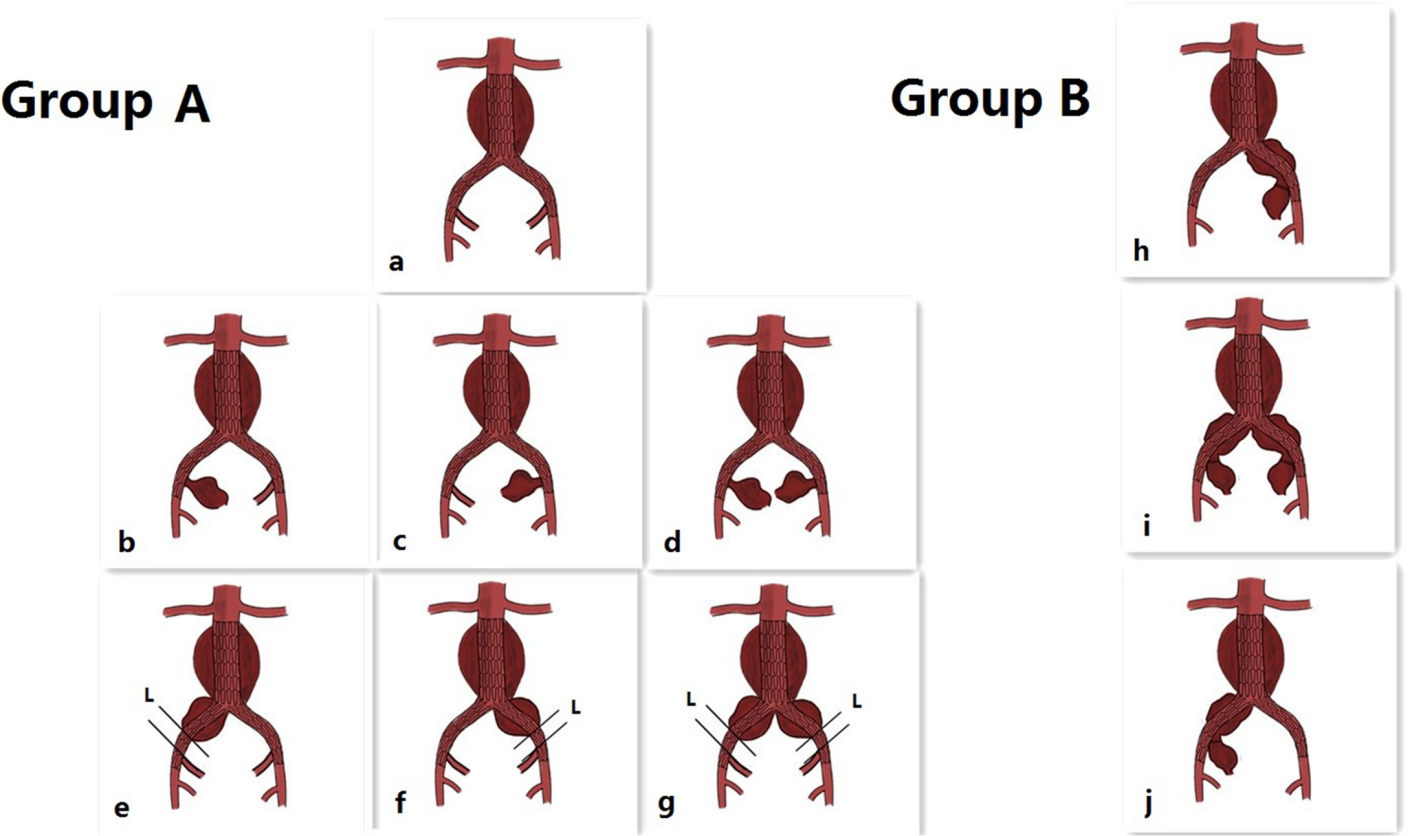
*Indications for group A (a, b, c, d, e, f, g): a: Standard abdominal aortic aneurysm (AAA; not coupled with common iliac artery aneurysm, CIAA), but the landing zone in the common iliac artery (CIA) was too short to anchor. b, c, d: Standard AAA coupled with internal iliac artery aneurysm (IIAA). e, f, g: AAA coupled with CIAA (unilateral or bilateral), and CIAA did not invade the original IIA, and the distance (L) from the distal end of the CIAA to the original IIA was less than 10–15 mm without stenosis or ectasia. *Indications for group B (h, I, j): AAA coupled with CIAA (unilateral or bilateral), and the CIAA invaded the original internal iliac artery (IIA). *Stents were supplied by Medtronic Inc (TALENT and ENDURANT series). Coils were supplied by Johnson & Johnson Company (Amplatzer).

1. Indications for IIA exclusion without embolization (Group A):
  1) Standard AAA [not coupled with common iliac artery aneurysm (CIAA)], but the landing zone in CIA was too short to anchor.
  2) AAA coupled with IIAA.
  3) AAA coupled with CIAA (unilateral or bilateral), CIAA did not invade the original IIA, and **L** (shown in Fig 1, the distance from the distal end of the CIAA to the original IIA) was less than 10–15 mm without stenosis or ectasia (a, b, c, d, e, f, g).

2 Indications for IIA exclusion with embolization (Group B):
  AAA coupled with CIAA that invaded the original IIA, and **L** was less than 10–15 mm (h, i, j).
  The Indications for IIA embolization are presented in Fig 1

#### Postoperative management after IIA exclusion

After EVAR, antiplatelet, anticoagulation and vasodilator therapies are essential for the patients (Aspirin, 100 mg/day; low molecular weight heparin, 600 mg/day; and PGE2, 40 μg/day); simultaneously, the skin temperature of the buttocks and limbs should be carefully checked, especially after IIA exclusion. If the skin temperature is lower than normal or tenderness and skin necrosis appear, reconstruction of the IIA should be considered in a timely manner.

Follow-up: After EVAR, discharged subjects were followed at the 1^st^, 3^rd^, 6^th^, and 12^th^ months, with annual visits thereafter for CT evaluations. The recorded endpoints included 30-day mortality, stent patency, and the occurrences of endoleaks and ischemic complications, such as ischemic colitis, spinal cord infraction, skin necrosis and chronic buttock claudication. The follow-up period ceased in June 2014.

#### Analysis Method

All the statistical analyses were performed using SPSS version 16 (SPSS Inc, Chicago, IL). The data are presented as the mean±standard deviation for continuous variables and as the frequency (percentage) for categorical variables, which were compared using the two-sample t-test, Fisher exact test, and Pearson Chi-square test where appropriate. Overall survival and patency curves were generated using the Kaplan-Meier method, and the log-rank test was used to compare the differences. Differences with a P value < .05 were considered to be significant.

### Ethics

This study was approved by the Ethics Committee of West China Hospital, Sichuan University. All the study participants provided written informed consent stating that the clinical data could be used in clinical research.

## Results

### Baseline comparisons

**Fig 2:**
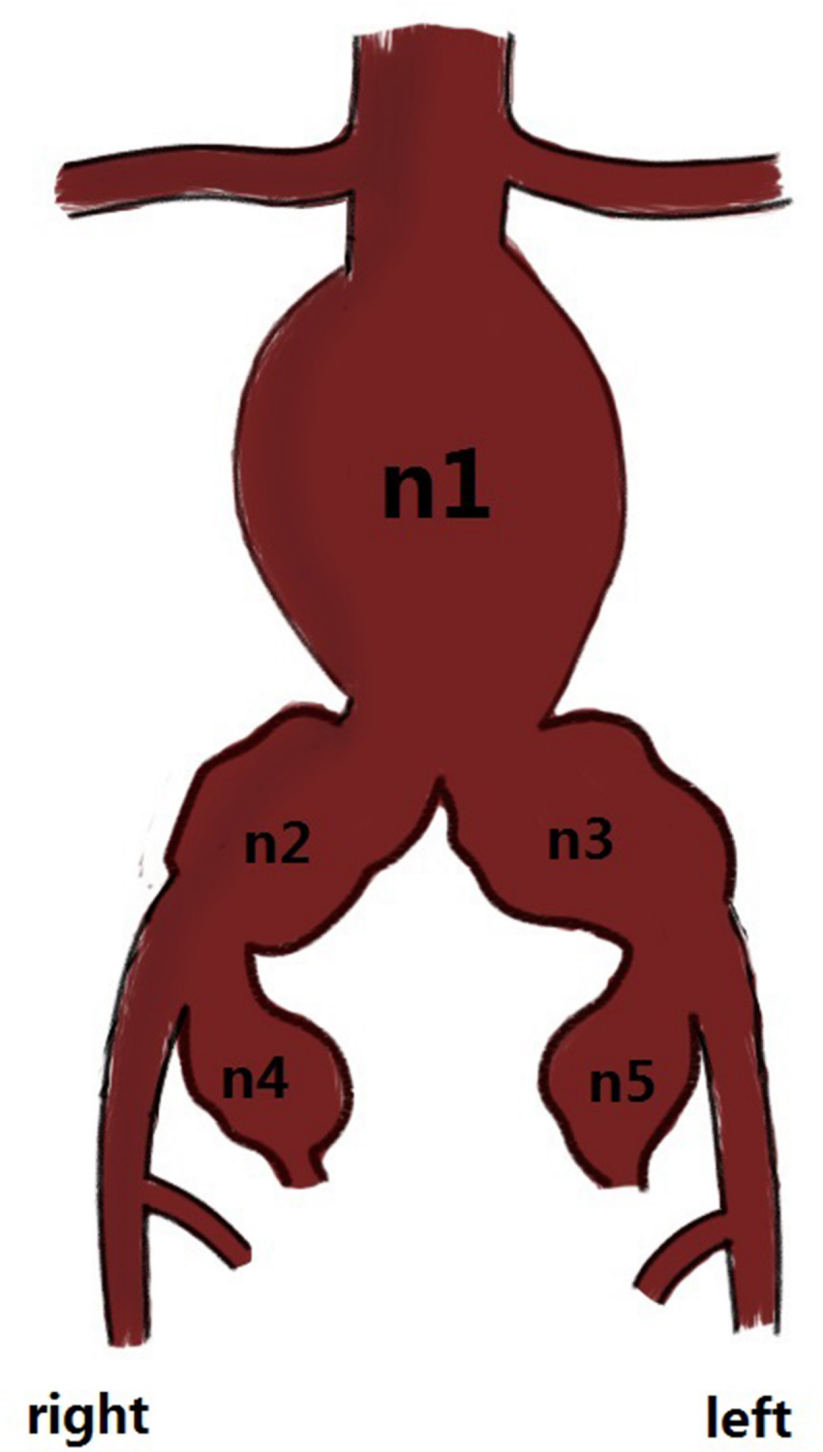
*n_1_: abdominal aortic aneurysm (AAA); n_2_: right common iliac artery aneurysm (CIAA); n_3_: left CIAA; n_4_: right internal iliac artery aneurysm (IIAA); and n_5_: left IIAA. *n_**1**_: 47; n_**1+2**_: 17; n_**1+3**_: 5; n_**1+4**_: 6; n_**1+5**_: 3; n_**1+2+3**_: 25; n_**1+2+5**_: 7; n_**1+2+4**_: 2; n_**1+3+4**_: 1; n_**1+3+5**_:1; n_**1+4+5**_: 3; n_**1+2+3+4**_: 4; n_**1+2+4+5**_: 2; n_**1+2+3+5**_: 5; and n_**1+2+3+4+5**_: 9. n_**1+2**_ represents an AAA that invaded the right common iliac artery; the others were considered likely. * Fig 2 is just an illustration; we cannot show the exact anatomical details.

Seventy-four subjects were in group A, and 63 subjects were in group B. Among the 137 consecutive subjects, 124 (91.1%) were male, and 13 (9.5%) were female, with mean ages of 71.56 and 71.67 years, respectively. The vascular morphologic characteristics of the 137 subjects are shown in Fig 2. General patient information is provided in Table 1.

**Table 1:** General information.

	Group A	Group B	P
Gender (male/female)	64/10	60/3	0.082
Age	71.43±6.88	73.06±6.94	0.823
Smoking history	58	47	0.603
Cardiovascular comorbidity	44	38	0.919
Respiratory comorbidity	16	14	0.932
Endocrine comorbidity	6	7	0.55
Renal comorbidity	1	2	0.594
Digestive comorbidity	8	9	0.539
Cerebrovascular disease	3	5	0.334
Carcinoma	6	0	0.021

Hypertension was defined as a pre-hemorrhage blood pressure documented as >140 mmHg systolic or >90 mmHg diastolic or the use of an anti-hypertensive agent. CAD and cerebral artery disease were defined by medical history or CTA of the coronary and cerebral artery, respectively. Respiratory failure was defined as an oxygen PaO2 greater than 80 mmHg (11 kPa) and/or a carbon dioxide PaCO2 less than 45 mmHg (6.0 kPa) or the need for intubation. Renal insufficiency was defined as serum creatinine ≥ 2 mg/dL. COPD, emphysema, bronchitis, and pulmonary bullae were mainly defined by medical history.

p<0.05 was considered statistically significant.

Anatomic parameter comparisons: aneurysm neck diameter: 21.4±3.3 mm (A: 21.5±2.9 mm vs B: 21.2±3.8 mm); aneurysm neck length: 25.1±7.9 (A: 25.5±7.5 mm vs B: 24.7±8.5 mm); diameter of aneurysm (anterior to posterior): 54.4±16.3 mm (A: 56.5±14.8 mm vs B: 51.8±17.8 mm); aneurysm length: 82.7±24.8 mm (A: 78.5±19.6 mm vs B: 86.9±28.9 mm); diameter of right CIA: 26.6±8.4 mm (A: 26.6±8.0 mm vs B: 26.6±9.1 mm); diameter of left CIA: 24.6±10.6 mm (A: 24.4±10.6 mm vs B: 24.7±10.6 mm); diameter of right IIA: 24.5±8.5 mm (A: 23.8±5.3 mm vs B: 26.5±14.8 mm); and diameter of left IIA: 25.9±6.8 mm (A: 26.1±4.2 mm vs B: 25.6±11.2 mm). Detailed anatomic baseline comparisons are provided in Table 2.

**Table 2:** 

	Group A	Group B	P
Diameter of neck	21.5±2.9	21.2±3.8	0.635
Length of neck	25.5±7.5	25.0±8.6	0.737
Diameter of aneurysm	56.5±14.7	51.8±17.8	0.110
Length of aneurysm	78.5±19.6	87.5±29.5	0.259
Diameter of left CIA	24.4±10.6	24.7±10.6	0.880
Diameter of right CIA	26.5±7.9	26.6±9.1	0.956
Diameter of left IIA	26.1±4.2	25.6±11.2	0.831
Diameter of right IIA	23.8±5.3	26.5±14.8	0.411

CIA: Common Iliac Artery; IIA: Internal Iliac Artery

mm: millimeter

p<0.05 was considered statistically significant.

### Early outcome comparison

All 137 patients underwent a successful EVAR, and there were no conversions to laparotomy. Fifty-four subjects (39.4%) received general anesthesia, 17 subjects (12.4%) had epidural anesthesia, and 66 subjects (48.2%) received local anesthesia. There was no significant difference in intraoperative blood loss (87.23±14.07 ml; A: 86.53±9.57 ml vs B: 88.06±18.04 ml, p = .545) or surgery time (87.13±9.25 min; A: 85.99±7.07 min vs B: 88.48±11.19 min, p = .130), and there were no blood transfusions during the operations. However, contrast consumption (65.18±9.85 ml; A: 61.89±7.95 ml vs B: 69.05±10.50 ml, p < .001) and intraoperative X-ray time (5.9±0.86 min; A: 5.63±0.49 min vs B: 6.22±1.07 min, P < .001) were significantly different. The 30-day mortality was approximately 0.73%. The early outcome comparisons are shown in Table 3.

**Table 3:** Early outcome comparisons between groups A and B.

	Group A	Group B	P
Anesthetics			
General	25	29	0.163
Epidural	10	7	0.797
Local	34	32	0.610
Intraoperative blood loss	86.53±9.57	88.06±18.04	0.545
Surgery time	85.99±7.07	88.48±11.19	0.130
Contrast consumption	61.89±7.95	69.05±10.50	< .001
Intraoperative X-ray time	50.63±0.49	60.22±1.07	< .001
30-day mortality	1	0	1.000

p<0.05 was considered statistically significant.

Intraoperative blood loss and contrast consumption were measured in milliliters, and surgery time and intraoperative X-ray time were measured in minutes.

### Outcome of follow-up

**Fig 3:**
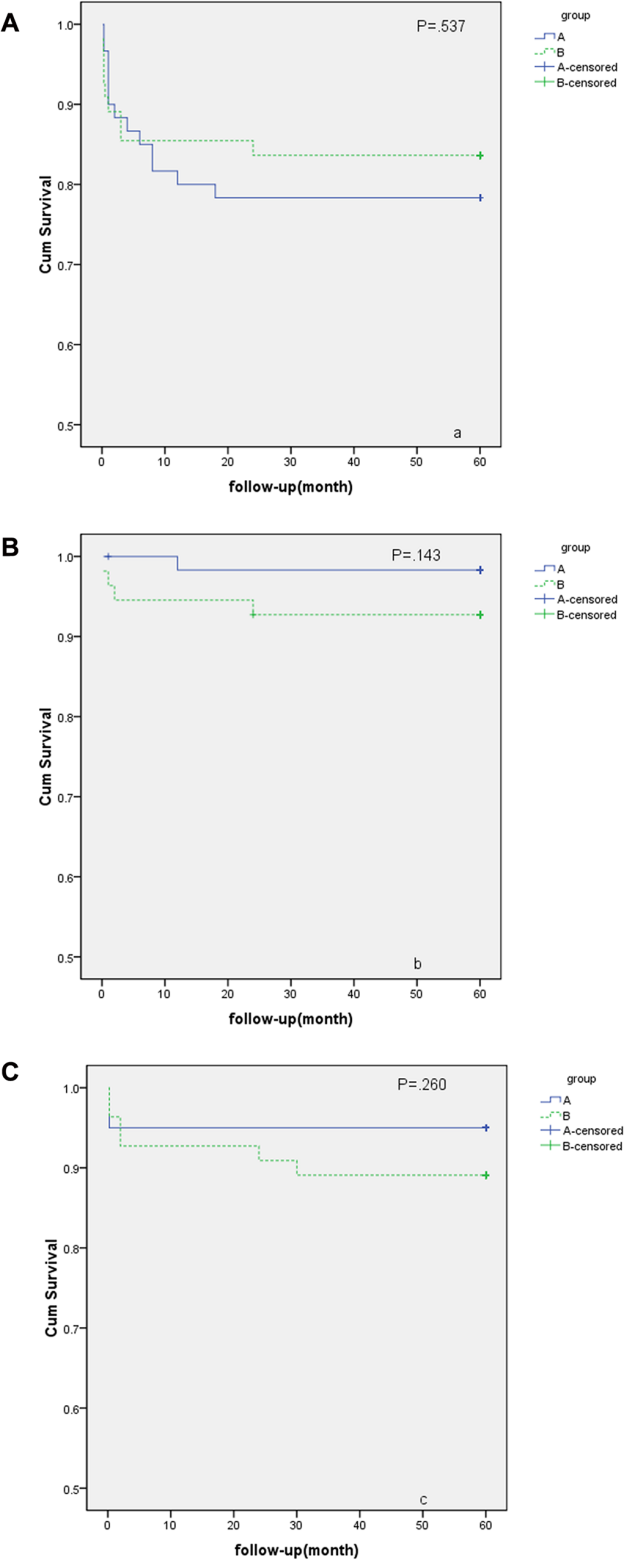
a: No significant difference was found in the survival analysis of endoleak between groups A and B (A: group A, B: group B) (P = .537). Internal iliac artery (IIA) coverage with embolization did not reduce the long-term risk of endoleak. b: No significant difference was identified in the survival analysis of patency between groups A and B (A: group A, B: group B) (P = .143). The incidence of occlusion during follow-up was not significantly different between groups A and B. c: No significant difference was found in the survival analysis of ischemic complications between groups A and B (A: group A, B: group B) (P = .260). However, the incidence of ischemic complications was higher in group B than in group A (A: 4.84% vs B: 11.11%), and more severe ischemic complications occurred in group B.

The study spanned 8 years, from June 2006 to June 2014, and the mean follow-up period was 61.2 months. No deaths or graft-associated deaths occurred during the follow-up period. Twenty-one patients were lost during follow-up, and 115 patients were followed. The overall incidences of endoleak, occlusion, and ischemic complications were 19.1%, 5.22%, and 4.35%, respectively. The comparisons of the incidences of endoleak, stent occlusion and ischemic complications between groups A and B during follow-up are shown in Table 4. The follow-up comparison between groups A and B is presented in Fig 3 (a, b, c).

**Table 4:** Incidences of complications in groups A and B.

	Total	Group A	Group B	P
Endoleak	22	13	9	0.647
Type I	4	2	2	1.000
Type II	12	8	4	0.666
Type III	7	4	3	1.000
Occlusion	5	1	4	0.180
Surgery	2	0	2	1.000
Conservative	3	1	2	1.000
Ischemic Complication	`9	3	6	0.301

p<0.05 was considered statistically significant.

Subjects underwent an additional operation after EVAR because of stent occlusion.

**Fig 4:**
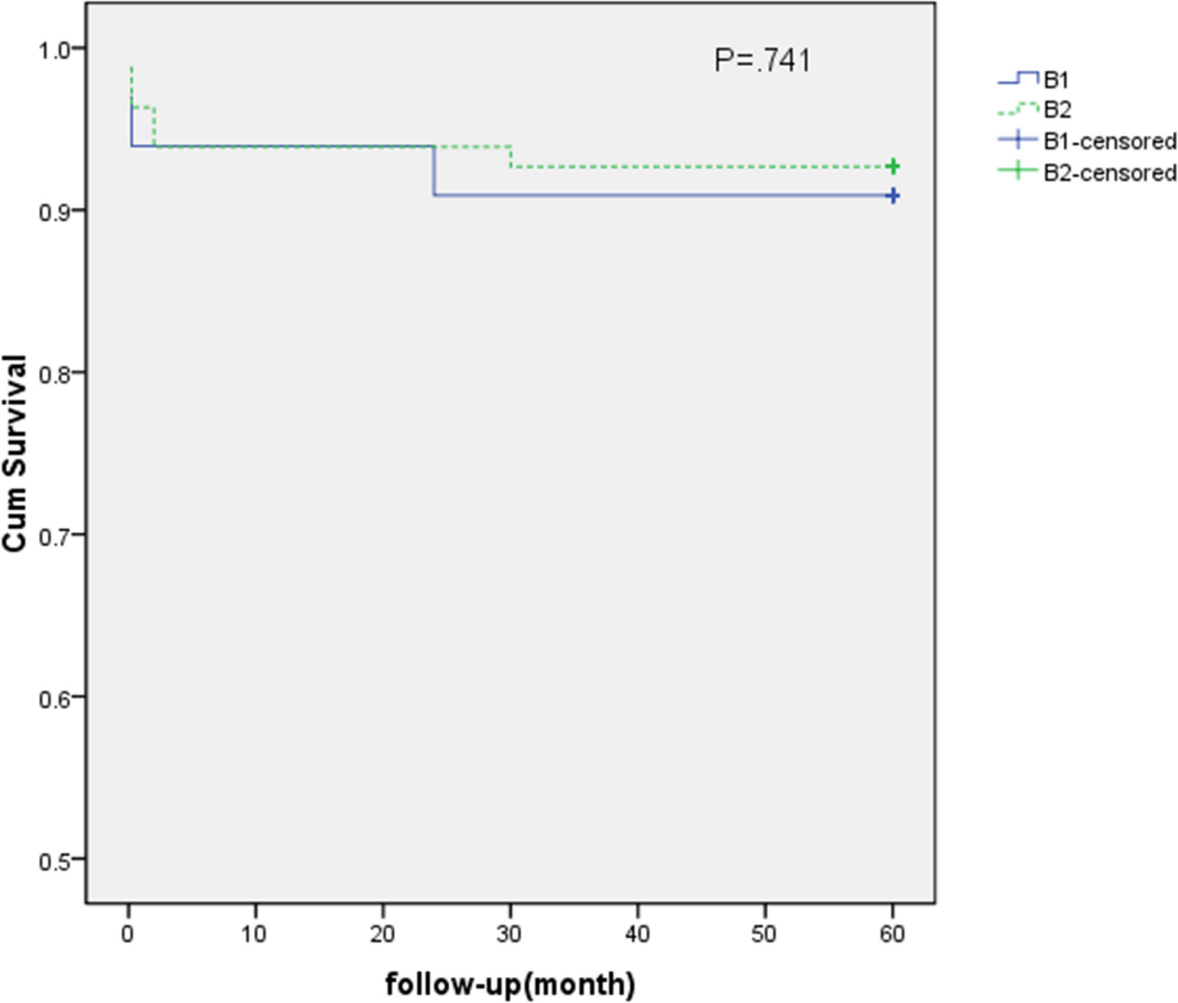
There was no significant difference in the incidence of postoperative ischemia between groups B1 (subjects with unilateral IIA exclusion, n = 4) and B2 (bilateral IIA exclusion, n = 2). However, B1 was obviously different from B2 in terms of hospital stays and the severity of the ischemic complications. The two subjects in B2 had hospital stays of 12 and 17 days; by contrast, the hospital stays of the subjects in B1 were 3, 5, 5, and 6 days (P < .001). A severe ischemic complication (gluteal skin necrosis) occurred in one subject in group B2 with a claudication distance of less than 100 meters. Gluteal soreness with a claudication distance of approximately 150 meters occurred in another subject in group B2. By contrast, gluteal ischemia and limb ischemia in group B1 were mild.

Endoleak occurred in 22 cases (A: 13 vs B: 9), with 4 Type I cases (A: 2 vs B: 2), 12 Type II cases (A: 8 vs B: 4), and 7 Type III cases (A: 4 vs B: 3). Type I and III endoleaks simultaneously occurred in one case. One subject in group A with a Type II endoleak underwent an endovascular intervention for an increased aneurysm and newly developed CIAA. In the other 21 cases, the endoleak disappeared or shrunk, and the size of the aneurysm did not increase during follow-up. Furthermore, the survival analysis revealed that there was no significant difference in the long-term incidence of endoleak between group A and B (P = .537; Fig 4A). Therefore, intraoperative embolization of the IIA with coils before coverage did not decrease the long-term incidence of type II endoleak.

Only 5 subjects (A: 1 vs B: 4) suffered from stent occlusion, and 2 symptomatic subjects underwent surgery (A: 0 vs B: 2, femoral-femoral bypass surgery and embolectomy surgery). Ischemic syndrome did not appear in the two patients thereafter. The 3 asymptomatic subjects (A: 1 vs B: 2) received conservative therapy. During conservative therapy, the ischemic symptoms were aggravated: the dermal temperature of the lower extremities decreased in the 2^nd^ month after EVAR and then recovered at the end of the 6^th^ month. Two other cases did not have any uncomfortable feelings during follow-up. The survival analysis revealed no significant differences between groups A and B in terms of stent occlusion (P = .143; Fig 4B), indicating that coverage with or without embolization does not affect long-term stent patency. Overall, the 5-year stent patency rate in our institution was 95.7%.

Ischemic complications, such as pelvic ischemia, spinal cord infraction, limb and gluteal ischemia, were a critical concern during follow-up. During the follow-up period, spinal cord infraction and pelvic ischemia were not observed in any subjects. Gluteal ischemia (A: 1 vs B: 4, P = 1.000) and limb ischemia (A: 2 vs B: 3, P = 1.000) after EVAR were observed in 9 subjects (A: 3 vs B: 6), and the incidence was 7.83%. One subject in group B simultaneously presented with buttocks claudication and limb ischemia and underwent a femoral-femoral bypass and embolectomy surgery 4 months after EVAR. Eight other subjects received conservative therapy. After 2–12 months of conservative therapy, ischemic syndrome was relieved in 8 cases. A comparison of the incidence of ischemic complications between groups A and B is shown in Table 5. Furthermore, no significant differences in ischemic complications between groups A and B were identified in the survival analysis (P = .26). Similarly, there were no significant differences in gluteal ischemia or limb ischemia based on the survival analysis. However, the incidence of ischemic complications was higher in group B than in group A (A: 4.84% vs B: 11.11%). Moreover, as shown in Fig 4C, there was a tendency towards chronic ischemia being more likely in group B and acute ischemia being more likely in group A. This trend will be discussed later in the discussion section.

**Table 5:** Ischemic complications and ischemia-related index comparisons.

	Group A	Group B	P
Ischemic Complication			
Claudication	3	6	0.301
Distance>400m	3	3	1.000
Distance<400m	0	3	0.095
Limb ischemia	2	3	0.661
Gluteal soreness	1	4	0.180
Skin and gluteal necrosis	0	1	0.460
Spinal cord infraction	0	0	/
Pelvic ischemia	0	0	/
Surgery	0	1	0.460
Amputation	0	0	/

p<0.05 was considered statistically significant.

The subgroup survival analysis (Fig 4) of group B suggested that there was no significant difference in the incidence of postoperative ischemia between groups B1 (subjects with unilateral IIA exclusion; n = 4) and B2 (bilateral IIA exclusion; n = 2). However, although there were no numerical differences between these subgroups, B1 obviously differed from B2 in terms of hospital stays and the severity of the ischemic complications. Two subjects in B2 stayed in the hospital for 12 and 17 days; by contrast, the patients in B1 stayed for 3, 5, 5, and 6 days (P < .001). A severe ischemic complication (gluteal skin necrosis) appeared in one subject in group B2 with a claudication distance of less than 100 meters. Gluteal soreness with a claudication distance of approximately 150 meters occurred in another subject in group B2. By contrast, gluteal ischemia and limb ischemia in group B1 were mild.

## Discussion

The incidence of AAA is increasing, and it is particularly obvious in the elderly. With the development of new techniques and increases in standards of living, more AAA patients would like to undergo an EVAR[3]. However, the fate of IIA remains disputable. It is controversial whether excluding the IIA with a coil is more effective at preventing type II endoleak after EVAR. Moreover, long-term results (greater than five years) are seldom reported but are crucial for clinical practice and guidance. Accordingly, this type of research is meaningful.

IIA exclusion may lead to gluteal and colonic ischemia and erectile dysfunction (ED)[7, 8]. Therefore, a comprehensive preoperative evaluation of blood supply in the lower extremities and buttocks is critical for surgery and prognostics, and IIA reconstruction must be performed when necessary, or postoperative ischemia is inevitable. In Dr. Rana’s research, freedom from buttock claudication was higher after open repair than after endovascular repair (79% vs 59%; P = 0.05), and reconstructions for IIA flow preservation had very good long-term patency. Every effort should be made to preserve IIA flow, as it leads to significantly better outcomes in terms of pelvic ischemic symptoms and buttock claudication[7].

In this study, there was no obvious significant difference in 5-year-long prognosis between group A (coverage without embolization) and group B (coverage with embolization). However, blood loss, surgery time, X-ray time, and contrast consumption were superior in group A compared to group B. Thus, patients in group A would likely receive a better long-term prognosis with less blood loss and contrast consumption as well as reduced surgery and X-ray times, which would be greatly beneficial for the patient. This likely conclusion was supported by Dr. Papazoglou’s research[12].

Furthermore, chronic ischemia rarely occurred in group A. A possible reason may be that the permeability of and unsolidified adherence to covered stents contributed to little blood flow into the IIA, leading to slow thrombosis in the IIA. Until a solidified embolism forms, there is enough time to establish collateral circulation, which ensures adequate blood supply to the lower extremities and buttocks. However, IIA coverage with embolization in group B caused fast, even acute, embolism that was not beneficial for the formation of collateral circulation, especially when bilateral IIA embolizations were performed. However, this tendency needs to be confirmed in future studies. If confirmed, it will be better to cover bilateral IIAs directly in staged surgery.

Dr. Lin[16] followed patients who underwent EVAR with unilateral or bilateral IIA exclusion and reported that 1) 50% of the patients in both groups (unilateral or bilateral IIA exclusion groups) experienced claudication; 2) scrotum desquamation occurred in 25% of the patients in the bilateral IIA exclusion group 3 days after EVAR, and a quarter of the patients experienced sacrococcygeal necrosis after surgery; and 3) ED occurred in 38% and 50% of the post-EVAR patients in the unilateral and bilateral groups, respectively. Dr. Johnston[17] collected and analyzed surgical records from 666 post-EVAR patients. The probability of colonic ischemia post-EVAR was 3% after bilateral IIA exclusion. By contrast, no patients experienced ischemia when at least one IIA was conserved. In this report of 75 followed cases with unilateral IIA exclusion (out of a total of 100), 6 (8.00%) presented with mild ipsilateral gluteal pain that worsened after walking with a claudication distance of approximately 350 m. In 31 followed cases with bilateral IIA exclusion (out of a total of 37), mild gluteal pain with a claudication distance of less than 200 meters occurred in 3 patients (9.68%); moreover, 1 patient suffered from severe ischemia (skin necrosis). Overall, ischemia appears more frequently in cases with bilateral IIA exclusion, and severe ischemia occurs more readily^3^.

Unfortunately, the evidence and outcome would be more powerful if this study was a randomized controlled trial (RCT) or included more participants. However, the results of our study are consistent with those of previous scientific studies, which encourages us to conduct a larger cohort study to confirm the current findings. In addition, the levels of the IIA exclusion with a coil were not exactly the same. Most IIAs were excluded at the trunk, which creates the lowest risk of ischemia, according to some research studies[18–20]. However, fewer than five subjects had exclusions at the branch level. Although some studies showed significant differences between patients with IIA embolization at different levels, the effect of the embolization level is negligible. If an iliac branch device (IBD) could be introduced into routine clinical practice in our hospital, it would be interesting to compare patients with and without IIA preservation. Interestingly, no significant differences in ischemic complications between patients with IIA embolization and with an IBD were reported in a recent study[21].

## Conclusion

In the traditional view, IIA embolization is an effective way to reduce the incidence of endoleaks, especially type II endoleaks. However, based on the present study, IIA coverage without embolization does not increase the long-term incidence of endoleak. Moreover, although there was no significant difference in ischemic complications with and without coil embolization, the differences in the severity of the ischemic complications and the reduced contrast consumption and X-ray time between the groups suggest that direct coverage is more effective and beneficial.
